# Anaesthesiologic Considerations for Intraoperative ECMO Anticoagulation During Lung Transplantation: A Single-Centre, Retrospective, Observational Study

**DOI:** 10.3389/ti.2024.12752

**Published:** 2024-03-20

**Authors:** Jaromir Vajter, Gabriela Holubova, Rene Novysedlak, Monika Svorcova, Jiri Vachtenheim, Tomas Vymazal, Robert Lischke

**Affiliations:** ^1^ Department of Anaesthesiology, Resuscitation, and Intensive Care Medicine, Second Faculty of Medicine, Charles University, University Hospital in Motol, Prague, Czechia; ^2^ Prague Lung Transplant Program, 3rd Department of Surgery, First Faculty of Medicine, Charles University, University Hospital in Motol, Prague, Czechia

**Keywords:** ECMO, UFH, anticoagulation, lung transplantation, anesthesiology

## Abstract

**Background:** Extracorporeal membrane oxygenation (ECMO) is frequently used during lung transplantation. Unfractionated heparin (UFH) is mainly used as part of ECMO support for anticoagulation. One of the most common perioperative complications is bleeding, which high-dose UFH can aggravate.

**Methods:** We retrospectively analyzed (*n* = 141) patients who underwent lung transplantation between 2020 and 2022. All subjects (*n* = 109) underwent central cannulated VA ECMO with successful intraoperative ECMO weaning. Patients on ECMO bridge, postoperative ECMO, heart-lung transplants and transplants without ECMO were excluded. The dose of UFH for the entire surgical procedure, blood loss and consumption of blood derivatives intraoperatively and 48 h after ICU admission were recorded. Surgical revision for postoperative bleeding were analyzed. Thrombotic complications, mortality and long-term survival were evaluated.

**Results:** Lower doses of UFH administered for intraoperative ECMO anticoagulation contribute to a reduction in intraoperative blood derivates consumption and blood loss with no thrombotic complications related to the patient or the ECMO circuit. Lower doses of UFH may lead to a decreased incidence of surgical revision for hemothorax.

**Conclusion:** Lower doses of UFH as part of intraoperative ECMO anticoagulation might reduce the incidence of complications and lead to better postoperative outcomes.

## Graphical Abstract

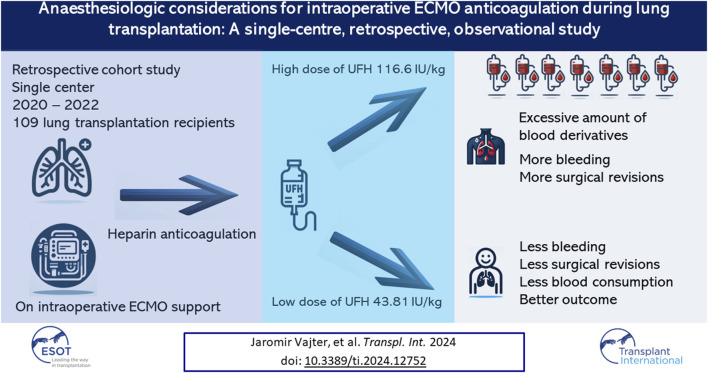

## Introduction

Intraoperative extracorporeal membrane oxygenation (ECMO) is routinely used during lung transplant surgery to provide the patient with temporary respiratory and circulatory support [1–3]. Over the past two decades, many centres have switched from intraoperative support with cardiopulmonary bypass (CPB) to ECMO [1, 2, 4, 5]. The intraoperative ECMO approach allows surgeons to perform the procedure with greater precision and efficiency while minimizing the risk of lung injury [2, 4]. Most centres that use intraoperative ECMO, to a greater extent, support the idea that ECMO helps to control lung graft reperfusion [4, 6, 7]. Another advantage of intraoperative ECMO support is the possibility of transplanting more patients with comorbidities who would not be able to undergo surgery on selective unilateral ventilation without extracorporeal support. For example, patients with severe pulmonary hypertension have a high risk of left-sided heart failure, pulmonary fibrosis, and very low pulmonary compliance [8, 9]. ECMO has a much lower pro-inflammatory potential than CPB [1]. However, ECMO also carries risks of bleeding, infection, and thrombotic complications [10–13]. The entire team should decide to use intraoperative ECMO on a case-by-case basis, considering the patient’s medical history and condition [14, 15]. Our study aims to highlight that lower doses of anticoagulants administered during intraoperative ECMO support do not lead to increased risks and may be beneficial for the patient.

### Intraoperative ECMO Anticoagulation During Lung Transplants

A certain amount of anticoagulation is crucial to prevent thrombotic complications during lung transplantation with intraoperative ECMO support [1, 2, 11]. Thrombotic events, such as clot formation, can cause significant harm to the patient and negatively impact the transplant’s success. However, the use of intraoperative anticoagulation is associated with a risk of bleeding, which can be problematic during surgery. Full heparinization with high activated clotting time (e.g., ACT >400 s) values are no longer necessary from CBP to ECMO support transition (also, thanks to heparin-coated ECMO cannulas and circuits). In contrast, the more knowledge we have about ECMO issues in the context of understanding coagulopathy, the more we strive for significantly lower doses of anticoagulation [4]. According to the current guidelines, the recommended procedure for ECMO cannulation is to administer a certain UFH bolus, usually 2000–5000 IU or 25–100 IU/kg, and then control the anticoagulation level using ACT [1, 11]. The ACT should be maintained in the range of 180–220 s [1, 11]. Other options (increasingly used over ACT) for anticoagulation monitoring are activated partial thrombin time (APTT) in the range of 60–90 s [16] and APTT ratio of 1.5–2.5 [16]. The anti-Xa assay is also a possible method for monitoring anticoagulation. The target values of the anti-Xa assay are 0.3–0.7 IU/mL [16]. According to the literature, it is also possible to perform anticoagulation monitoring using viscoelastic methods such as rotational thromboelastometry (ROTEM), explicitly using the CT INTEM/HEPTEM ratio [17].

## Materials and Methods

This study was approved by the Local Ethics Committee (reference number EK-786/23) and was registered in the clinical trial database at ClinicalTrials.gov (identifier number NCT06054997). This study was designed as a single-centre, retrospective, observational study that included all lung transplants performed between January 2020 and December 2022 within the Prague Lung Transplant Program Motol University Hospital. A total of 141 patients underwent transplantation during the study period. The exclusion criteria were lung transplantation without intraoperative ECMO support, block heart-lung transplantation, ECMO bridge-to-lung transplant, and planned postoperative ECMO. The inclusion criteria were lung transplantation performed with intraoperative ECMO support, central ECMO cannulation, and successful ECMO support termination at the end of surgery. According to the study inclusion criteria, only lung transplants performed under intraoperative central ECMO cannulation with successful ECMO weaning were included. In total, 109 patients fulfilled the inclusion criteria. Thirty-two patients were excluded based on the following criteria: heart-lung transplant (HLTx), *n* = 4; ECMO bridge, *n* = 4; transplantation (Tx) without ECMO, *n* = 8; and planned prolonged ECMO, *n* = 16. UFH was used for ECMO anticoagulation in all the patients. The subjects were divided into two groups for further analysis according to the UFH dose administered during the entire surgical procedure. In the first group, the UFH dose was ≤60 IU/kg/surgery. In the second group, the UFH dose was greater than 61 IU/kg/surgery. A cutoff value of 60 IU/kg was determined based on the available literature review. Values ≤60 IU/kg/surgery were considered relatively lower doses, and values >61 IU/kg/surgery were considered higher doses of UFH [2, 4, 10–13, 16–19]. The UFH effect was monitored using activated clotting time (ACT) values. The intraoperative haemoglobin level target for red blood cell (RBC) substitution was 100 g/L. Coagulopathy was managed according to the clinical experience of the anesthesiologist and viscoelastic Point of care methods (ROTEM, PFA). The parameters followed up intraoperatively in both groups were total blood loss in milliliters and related to the patient’s weight during the surgery (assessed by the amount of blood in a calibrated suction device); the total amount of UFH administered to the patient during surgery in the international unit (IU) and related to patient weight; and the consumption of blood derivatives during the surgical procedure, such as RBC, fresh frozen plasma (FFP), and platelets (PLT). In both groups, ACT values were monitored after the administration of UFH before ECMO cannulation and then every 60 min. In both groups, protamine was administered at the end of the surgical procedure until physiological ACT values below 120 s were achieved (if needed). No type of biological glue to seal the anastomosis was used. In both groups, intraoperative VA ECMO was implanted electively, and no patient underwent urgent ECMO cannulation due to cardiac or pulmonary reasons. The Maquet Rotaflow RF-32 centrifugal pump provided Intraoperative VA ECMO support. Heparin-coated cannulas and a heparin-coated tubing system were used for cannulation. According to internal guidelines, the ECMO flow was maintained at 1/2 to 2/3 of the calculated cardiac output. In the postoperative period, we followed up on the development of hemothorax requiring surgical revision. We considered surgical revision for hemothorax to be a significant bleeding complication. We also evaluated blood product consumption in the period 48 h after ICU admission, PGD grade three in 72 h after LUTx, 30-day and 90-day mortality, long-term survival and ECMO circuit-related and patient-related thrombotic complications. The basic, standard points of care in the Intensive care unit (ICU) are displayed in Table 1.

**TABLE 1:** Standard, essential post-transplant ICU care.

Mechanical ventilation
• Maximum effort to achieve early extubation
• Upon admission to the Intensive Care Unit
• Pressure control mode mechanical ventilation (initially PEEP 8–10 cmH_2_O)
• Pressure support mode mechanical ventilation (PEEP 5 cmH_2_O)
• extubation
Vasopressoric support
• First choice—Norepinephrine
• Additional—Vasopressin
Immunosuppressives medication
• Tacrolimus
• Mycophenolate-mofetil
• Methylprednisolone
• Basiliximab
Antibiotics and antivirotics
• Piperaciline-Tazobactam i.v.
• Amphotericin inh.
• Ganciclovir i.v.
Analgesia
combination, according to a visual analogue scale and patient needs
• Bilateral ESPB
• Sufentanil
• Paracetamol
• NSAID
• Dexmedetomidine
• LMWH (Enoxaparine) s.c. based on antiXa assay
• Ketamin
Thrombosis prevention
• LMWH (Enoxaparine) s.c. based on antiXa assay
Specific examination and procedures
• Chest X-ray every 24 h
• Physiotherapy 4 times/day or based on the needs of the patient
• Microbiology findings every 24 h
• Pulmonary artery pressure continual monitoring
• Continual hemodynamic monitoring
• Early enteral feeding

Abbreviations: PEEP, Positive end-expiratory pressure; cmH_2_O centimeter of water; i.v. intravenous; inh. inhalation; NSAID, non-steroidal anti-inflammatory drugs; LMWH, low molecular weight heparin, s.c. subcutaneous.

### Recipient and Donor Characteristics

Both groups of recipients were relatively homogenous, even though the number of subjects was not the same in both groups (lower dose UFH/kg group, *n* = 44; higher dose UFH/kg group, *n* = 65). The *p*-values for sex, age, height and mean pulmonary artery pressure (mPAP) were above the significance level of 0.05. The lower dose UFH/kg group recipients had a slightly higher weight (*p* = 0.048) and a higher BMI (*p* = 0.0093). The distribution of diagnoses for which the recipients were transplanted was also homogeneous (*p* > 0.05). These numbers are listed in Table 2. Donor characteristics were completely homogeneous in both groups. The *p*-values of sex, age, weight, height, BMI, and cause of death were above the significance level of 0.05 (Table 2).

**TABLE 2:** Recipient and donor characteristics.

Recipient-characteristic variable	≤60 IU/kg UFH group (*n* = 44)	>61 IU/kg UFH group (*n* = 65)	*p*-value
Male sex, n (%)	26 (59.09%)	44 (67.69%)	0.36
Age (years; mean ± SD)	54.14 ± 11.53	52.09 ± 12.47	0.59
Weight (kg; mean ± SD)	79.91 ± 15.47	73.93 ± 15.26	0.048
Height (cm; mean ± SD)	172.1 ± 7.39	173.4 ± 7.72	0.39
Body mass index (mean ± SD)	26.9 ± 4.43	24.6 ± 4.57	0.0093
mPAP (torr; mean ± SD)	26.3 ± 9.6	27.8 ± 13.4	0.528
thoracotomy prior LUTx (sum)	1	1	0.78
Transplant indication			
COPD, n (%)	15 (34%)	26 (40%)	0.59
Pulmonary fibrosis, n (%)	18 (40.9%)	23 (35.38%)	0.50
Cystic fibrosis, n (%)	3 (6.8%)	5 (7.69%)	0.89
Others, n (%)	8 (18.18%)	11 (16.92%)	0.82

Donor-characteristic variable	≤60 IU/kg UFH group (*n* = 44)	>61 IU/kg UFH group (*n* = 65)	*p*-value
Male sex, n (%)	21 (41.72%)	37 (56.92%)	0.34
Age (years; mean ± SD)	47.41 ± 16.05	44.78 ± 16.32	0.41
Weight (kg; mean ± SD)	70.9 ± 13.62	76.7 ± 17.12	0.059
Height (cm; mean ± SD)	170.3 ± 10.12	172.6 ± 10.95	0.26
Body mass index (mean ± SD)	24.1 ± 3.64	25.6 ± 4.7	0.074
Cause of death			
Subarachnoid haemorrhage, n (%)	10 (22.7%)	14 (21.5%)	0.88
Intracerebral bleeding, n (%)	8 (18.2%)	14 (21.5%)	0.67
Traumatic brain injury, n (%)	15 (34.1%)	16 (24.6%)	0.28
Anoxic brain injury, n (%)	8 (18.2%)	13 (20.0%)	0.81
Others, n (%)	3 (6.8%)	8 (12.3%)	0.35

Abbreviations: IU, international unit; UFH, unfractionated heparin; SD, standard deviation; n, Number of Subjects; kg, Kilogram(s); COPD, chronic obstructive pulmonary disease; mPAP, mean pulmonary arterial pressure.

### Statistical Analysis

Statistical analyses were performed with version 8.0.1 (244) of GraphPad Prism statistical software. Statistical significance was set at *p* < 0.05. The unpaired *t*-test was used to statistically evaluate blood loss, UFH dose, consumption of blood derivatives, and ACT values. For the statistical evaluation of surgical revision for hemothorax and PGD, we chose chi-square test. Kaplan-Mayer curve and log-rank test have been performed for 30-day and 90-day” and long-term survival assessment (long-term survival time endpoint October/2023).

## Results

**FIGURE 1:**
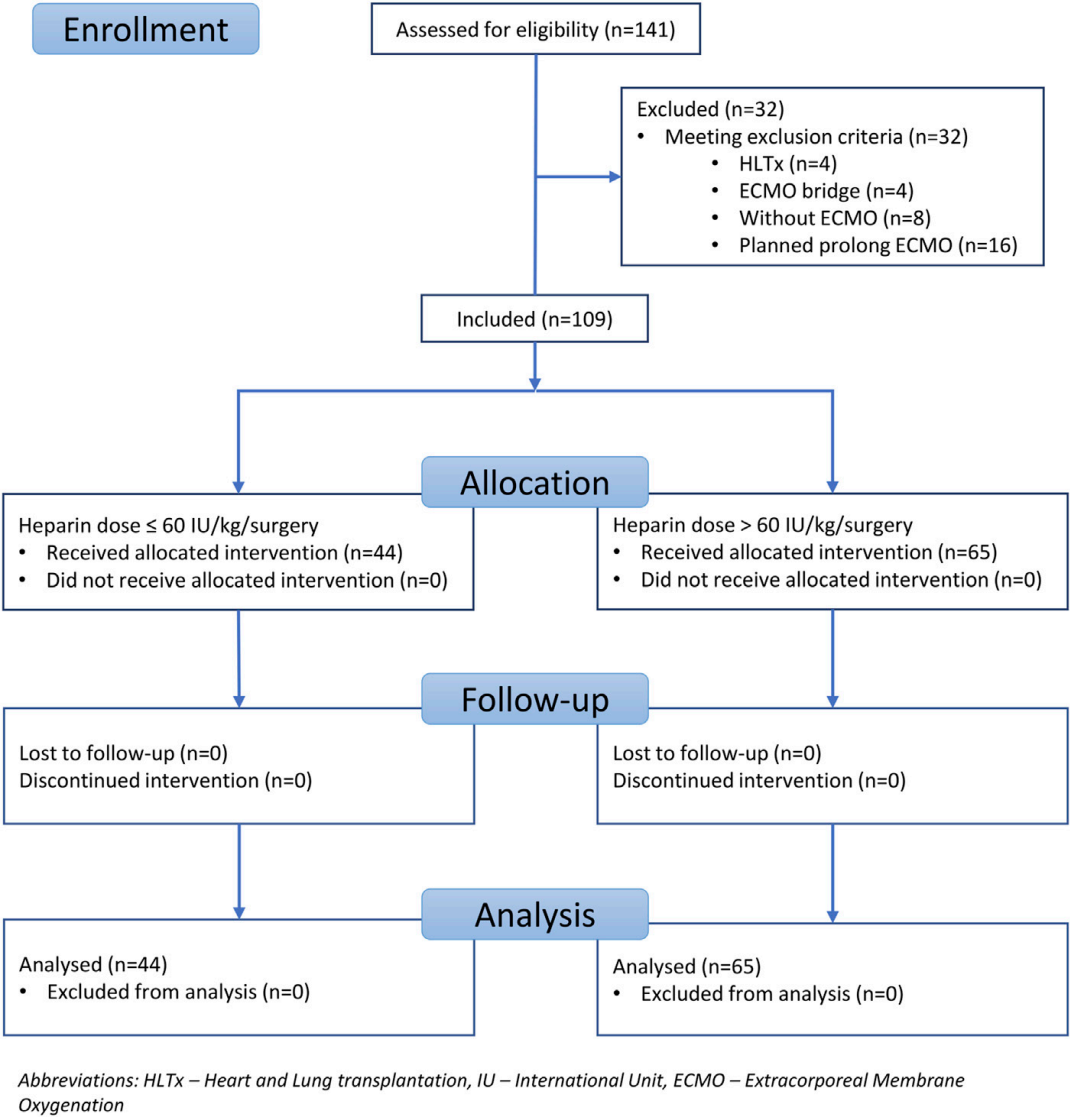
Flow chart of the study population.

**FIGURE 2:**
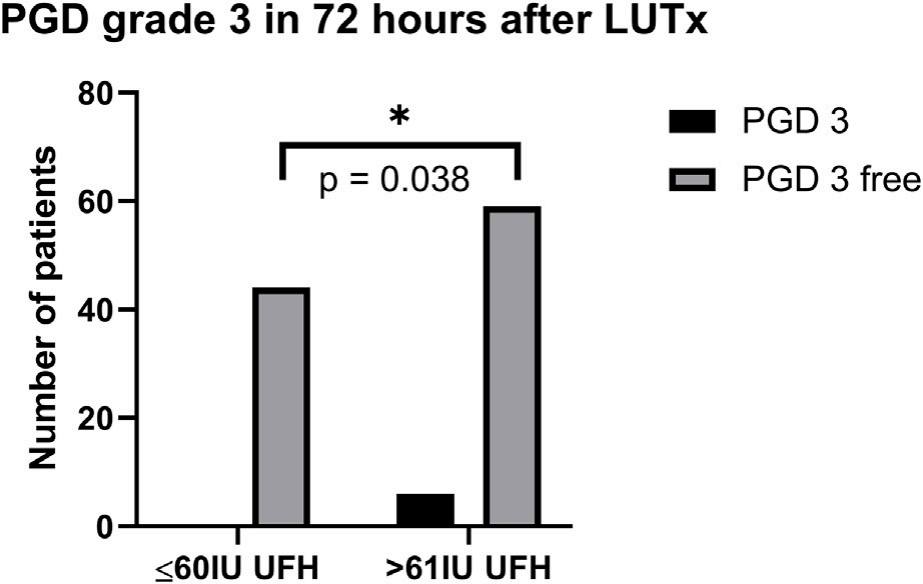
PGD grade 3 in 72 h after LUTx. Abbreviations: PGD, primary graft dysfunction; UFH, unfractionated heparin; LUTx, lung transplantation.

**FIGURE 3:**
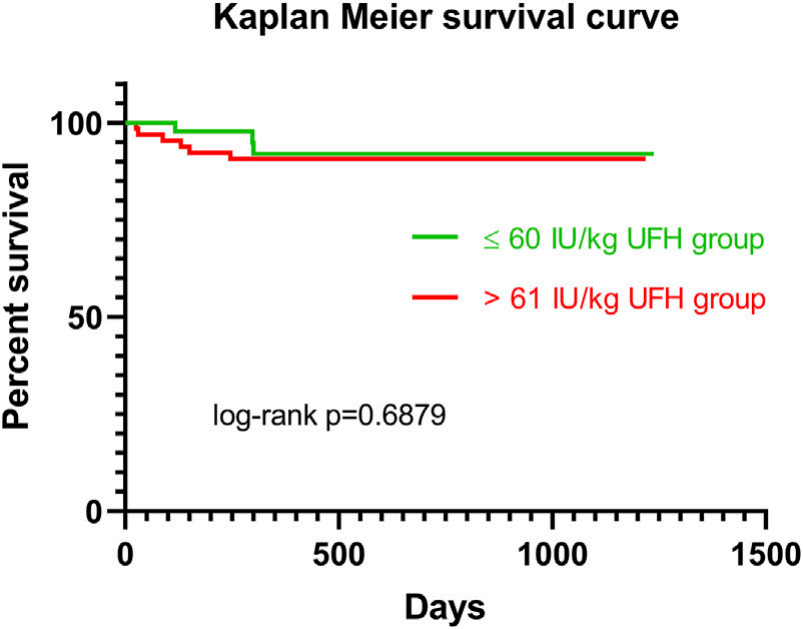
Kaplan Meier survival curve for patients in ≤60 IU/kg UFH group (green line) vs >61 IU/kg UFH group (red line). Abbreviations: IU, international unit; UFH, unfractionated heparin.

Patients were recruited between January 2020 and December 2022, and based on the exclusion criteria, a total of 32/141 patients were excluded from the study. A flow diagram based on the Consolidated Standards of Reporting Trials (CONSORT) is shown in Figure 1. The lower dose of UFH (≤60 IU/kg/surgical procedure) and higher dose of UFH (>61 IU/kg/surgical procedure) groups ultimately consisted of 44 and 65 patients, respectively. For most parameters, we obtained surprising results, clearly in favour of the administration of lower doses of UFH (≤60 IU/kg). Total blood loss during surgery was significantly lower in the group treated with lower doses of UFH (≤60 IU/kg). The mean total intraoperative blood loss was 753 and 1,470 mL, respectively (*p* < 0.0001) (Table 3). Blood loss related to body weight was also significantly lower in the UFH group ≤60 IU/kg). The mean intraoperative blood loss/patient body weight was 9.628 mL/kg and 20.97 mL/kg, respectively (*p* < 0.0001) (Table 3). The total UFH dose was significantly lower in the UFH group (≤60 IU/kg). The mean total intraoperative UFH doses were 3491 IU and 8694 IU, respectively (*p* < 0.0001) (Table 3). The total UFH dose, based on body weight, was significantly lower in the UFH group (≤60 IU/kg). The mean dose of UFH/patient bodyweight intraoperatively was 43.81 IU/kg and 116.6 IU/kg, respectively (*p* < 0.0001) (Table 3). We also noticed a significant difference in favour of reducing the consumption of blood derivatives in the group with lower doses of UFH ≤60 IU/kg. The consumption of RBCs during the surgical procedure was 0.5581 and 1.908 units, respectively (*p* = 0.0009) (Table 3). The FFP consumption during surgery was 0.4186 and 1.862 units, respectively (*p* = 0.0009) (Table 3). The platelet consumption during surgery was 0.1628 and 0.4154 units, respectively (*p* = 0.1461) (Table 3). The mean ACT values before ECMO cannulation and 3 min after the administration of UFH were lower in the UFH group ≤60 IU/kg (156.3 and 209.1) (*p* < 0.0001) (Table 3). There was a significant reduction in bleeding complications in terms of surgical revision for hemothorax in the lower UFH dose group ≤60 IU/kg, with only one revision (2.27%) and nine revisions (13.85%), respectively (*p* = 0.040) (Table 3). In the postoperative period 48 h after admission to the ICU, we did not observe a significant decrease in the consumption of blood derivatives (Table 4). However, there was a significantly lower incidence of third-degree PGD 72 h after LUTx in the group where a lower dose of UFH was administered (*p* = 0.038) (Table 4; Figure 2). The 30-day, 90-day, and long-term survivals were not different (Figure 3). The log-rank test was *p* = 0,6879 (Figure 3). Mortality rates were not different in either group. We did not record any thrombotic complications arising from the ECMO circuit or patient-related complications in any group.

**TABLE 3:** Variables blood loss, intraoperative blood product consumption, UFH dose, and surgical revision for haemothorax.

Variables	≤60 IU/kg UFH group (*n* = 44)	**> 61 IU/kg UFH group (*n* = 65)**	*p*-value
**Total blood loss (ml; mean ± SD)**	753.5 ± 522.5	1,470 ± 1,014	**<0.0001**
**Blood loss per kg (ml/kg; mean ± SD)**	9.63 ± 6.59	20.97 ± 15.64	**<0.0001**
**Total dose of heparin (IU; mean ± SD)**	3,491 ± 1,088	8,694 ± 3,790	**<0.0001**
**Dose of heparin per kg (IU/kg; mean ± SD)**	43.81 ± 12.08	116.6 ± 44.98	**<0.0001**
**RBC consumption (unit)**	0.5581 ± 1.119	1.908 ± 2.435	**0.0009**
**FFP consumption (unit)**	0.4186 ± 1.349	1.862 ± 2.543	**0.0009**
**PLT consumption (unit)**	0.1628 ± 0.5314	0.4154 ± 1.044	0.1461
**Surgical revision due to haemothorax (% of revision)**	2.27	13.85	**0.040**

Abbreviations: IU, international unit; UFH, unfractionated heparin, mL, millilitre; kg, kilogram; SD, standard deviation; RBC, red blood cells; FFP, fresh frozen plasma; PLT, platelets.

Bold values are statistically significant.

**TABLE 4:** Variables post LUTx (PGD 3 in 72hours post LUTx), (FFP, RBC, PLT 48 h post LUTx).

Variables	≤60 IU/kg UFH group (*n* = 44)	**> 61 IU/kg UFH group (*n* = 65)**	*p*-value
**PGD grade 3 (total, percentage)**	**0 (0%)**	**6 (9.23%)**	**0.038**
**RBCs consumption mean (unit)**	1.43 ± 2.0	1.55 ± 2.4	0.7996
**FFP consumption mean (unit)**	0.41 ± 1.4	1.12 ± 3.0	0.1567
**PLT consumption mean (unit)**	0.23 ± 1.1	0.35 ± 1.1	0.5615

Abbreviations: IU, international unit; UFH, unfractionated heparin; LUTx, lung transplantation; PGD, primary graft dysfunction; FFP, fresh frozen plasma; RBCs, red blood cells; PLT, platelets; IU, international unit.

Bold values are statistically significant.

## Discussion

Intraoperative ECMO support during lung transplantation is a routine method frequently used to facilitate surgical procedures. This enables the procedure to be performed in significantly more polymorbid patients who cannot handle selective ventilation. Another indisputable advantage of ECMO is the possibility of controlled reperfusion of lung grafts. During ECMO support, the blood is in contact with allogeneic materials such as cannulas, circuits, and oxygenators. All of these factors can cause potential complications, including bleeding and thrombosis. Therefore, balancing the edge between anticoagulation and procoagulation is very important to minimize complications during intraoperative ECMO support. UFH remains the most widely used drug for ECMO anticoagulation. From the available recommendations of thoracosurgery societies, we know the recommendation for UFH dosing, most often between 25–100 IU/kg and effect control with ACT in the range of 180–220 s. However, the literature and the guidelines more frequently report the possibility of reducing the dose of UFH and, very importantly, without an increase in thrombotic complications. According to the recommendations of the thoracosurgery societies, in the case of bleeding complications or anticipated intraoperative bleeding (for example, significant intrapleural adhesions), it is recommended to minimize the dose of UFH or completely eliminate UFH and perform heparin-free ECMO. For example, Bernhardt et al. mentioned in The International Society for Heart and Lung Transplantation/Heart Failure Society of America Guidelines on Acute Mechanical Circulatory Support that “bleeding complications in acute mechanical circulatory support (MCS) are common and frequently necessitate withdrawal of anticoagulation” and stated that “in the settings of life-threatening bleeding, full discontinuation of all anticoagulation may be necessary” [20]. Hartwig et al. stated in The American Association for Thoracic Surgery guidelines that “low or no heparin regimes are recommended for patients with significant adhesions and impaired coagulation status” [1]. Additionally, Lorusso et al., in the EACTS/ELSO/STS/AATS expert consensus, mentioned that “anticoagulation is required during prolonged ECLS to prevent circuit thrombus formation with embolization and/or circuit failure. However, bleeding remains the most frequent complication associated with ECLS. UFH infusion is typically delayed until haemostasis is achieved, often within 24–48 h. Reports that suggest the safety of prolonged withdrawal of anticoagulation for as long as 3 days when faced with bleeding are important” [21]. This trend was also confirmed by our study, which suggests that a lower dose of UFH as part of intraoperative ECMO support may not pose a risk but, on the contrary, can have substantial benefits for the patient. Using lower doses of UFH below 60 IU/kg (43.81 IU/kg based on our study) can benefit the patient and the entire perioperative period. Nonetheless, these results must be interpreted with caution, and numerous limitations should be considered. The major limitation is that the dosage of the UFH used in the study (high or low dose) has been based on subjective criteria, i.e., the clinical experience of the anesthesiologist guides its dosage. Another possible limitation of this study may be the division of subjects according to UFH into groups below and above 60 IU/kg since the available literature does not define the exact dose of UFH but only the range. Therefore, it was necessary to set a limit for dividing the patients into groups. However, the medians of the two groups were very far apart 43.81 IU/kg vs. 116.6 IU/kg; we presume this is more of a minor bias. Furthermore, having a more significant number of investigated subjects would be advisable, which would add even more weight to the entire study. Similarly, a particular bias may have been introduced into this study because the primary hemostasis disorder was not investigated; primary hemostasis can be disturbed as part of ECMO support during lung transplantation, thereby potentiating intraoperative bleeding [22]. The authors hypothesize that the significantly higher incidence of PGD grade 3 in 72 h postop in the group with higher doses of UFH may be caused by the need to administer a larger number of blood derivatives, which is one of the possible reason for the development of PGD. The anesthesiologist plays a crucial role in the decision-making process and is mostly responsible for anticoagulation management of intraoperative ECMO support [15, 23, 24]. Such management depends not only on their experience but also on their knowledge of the latest findings and recommended practices. The decision to withhold anticoagulation therapy or decrease its dosage involves balancing the competing risks between bleeding and clotting.

### Conclusion

The results of this study generates a hypothesis that lower doses of UFH (mean dose of UFH: 43.81 IU/kg) administered for intraoperative ECMO anticoagulation may contribute to a reduction in intraoperative blood loss and decrease the incidence of surgical revisions for haemothorax. Furthermore, lower doses of UFH may reduce the intraoperative consumption of blood derivatives such as RBCs and FFP. Notably, the concept of lower doses of UFH did not have a negative effect on 30-day, 90-day and long-term survival. No thrombotic complications of the ECMO circuit or thrombotic complications related to the patient were observed. Further investigation in this area is needed to provide deeper insights into the potential use of lower doses of UFH during ECMO.

## Data Availability

The raw data supporting the conclusion of this article will be made available by the authors, without undue reservation.

## Abbreviations

ACT, activated clotting time; APTT, activated partial thrombin time; BMI, body mass index; CONSORT, Consolidated Standards for Reporting Trials; CPB, cardiopulmonary bypass; ECMO, extracorporeal membrane oxygenation; FFP, fresh frozen plasma; HLTx, heart-lung transplant; ICU, intensive care unit; LUTx, lung transplant; MCS, mechanical circulatory support; NSAID, non-steroidal anti-inflammatory drugs; PEEP, positive end-expiratory pressure; PFA, platelet function assay; RBCs, red blood cells; ROTEM, rotational thromboelastometry; Tx, transplantation; UFH, unfractionated heparin.
